# Spatial accessibility to specific sport facilities and corresponding sport practice: the RECORD Study

**DOI:** 10.1186/1479-5868-10-48

**Published:** 2013-04-20

**Authors:** Noëlla Karusisi, Frédérique Thomas, Julie Méline, Basile Chaix

**Affiliations:** 1 Inserm, U707, Faculté de Médecine Saint-Antoine, 27 rue Chaligny, 75012 Paris, France; 2 Université Pierre et Marie Curie-Paris6, UMR-S 707, Faculté de Médecine Saint-Antoine, 27 rue Chaligny, 75012 Paris, France; 3 Centre d’Investigations Préventives et Cliniques, 6 rue La Pérouse, 75116 Paris, France

**Keywords:** Sports facilities, Spatial accessibility, Physical activity, Neighborhood factors

## Abstract

**Background:**

Physical activity is considered as a major component of a healthy lifestyle. However, few studies have examined the relationships between the spatial accessibility to sport facilities and sport practice with a sufficient degree of specificity. The aim of this study was to investigate the associations between the spatial accessibility to specific types of sports facilities and the practice of the corresponding sports after carefully controlling for various individual socio-demographic characteristics and neighborhood socioeconomic variables.

**Methods:**

Data from the RECORD Study involving 7290 participants recruited in 2007–2008, aged 30–79 years, and residing in the Paris metropolitan area were analyzed. Four categories of sports were studied: team sports, racket sports, swimming and related activities, and fitness. Spatial accessibility to sport facilities was measured with two complementary approaches that both take into account the street network (distance to the nearest facility and count of facilities around the dwelling). Associations between the spatial accessibility to sport facilities and the practice of the corresponding sports were assessed using multilevel logistic regression after adjusting for individual and contextual characteristics.

**Results:**

High individual education and high household income were associated with the practice of racket sports, swimming or related activities, and fitness over the previous 7 days. The spatial accessibility to swimming pools was associated with swimming and related sports, even after adjustment for individual/contextual factors. The spatial accessibility to facilities was not related to the practice of other sports. High neighborhood income was associated with the practice of a racket sport and fitness.

**Conclusions:**

Accessibility is a multi-dimensional concept that integrates educational, financial, and geographical aspects. Our work supports the evidence that strategies to increase participation in sport activities should improve the spatial and financial access to specific facilities, but also address educational disparities in sport practice.

## Background

Regular physical activity is known to prevent chronic diseases such as obesity, diabetes, and some cancers [1-3]. Previous studies on the effects of residential environments on physical activity have highlighted the positive influence that the presence of sport facilities may have on people’s physical activity behavior. Accordingly, the spatial accessibility to sport facilities is a major issue for many types of stakeholders in policy making in the fields of transport, urban planning, marketing, and public health [4-8]. A limited body of research has examined associations between the spatial availability to facilities as assessed through objective (rather than self-reported) measures and physical activity behavior [9-12].

Despite overall satisfactory levels of spatial accessibility to sports facilities in the Paris metropolitan area, previous work showed significant differences in the types of facilities available in the various neighborhoods, especially between advantaged and disadvantaged neighborhoods [13]. It is therefore important to examine whether these disparities in spatial accessibility to specific types of facilities have an impact on the practice of the corresponding sports.

However, most studies that examined relationships between spatial accessibility to sport facilities and physical activity or sport practice have taken into account overall measures of accessibility combining facilities of various types and overall measures of physical activity combining various sports or recreational and non-recreational activity [9,10,12,14-19]. One study derived separate spatial accessibility variables for various types of sport facilities but correlated these variables with an overall measure of physical activity [11]. Other studies only distinguished public from private facilities [10] or parks from other facilities [9,12,19] and therefore relied on information on facilities with a limited degree of specificity. One study from Spain correlated densities of swimming pools and gyms with the use of the corresponding facilities, but the analyses were conducted at a very broad area level (provinces) [20]. The absence or the weakness of associations reported may be due to the lack of specificity of the associations examined and to the mix of heterogeneous sport activities. To address this limitation, our strategy was to increase the specificity of the associations examined (i.e., by focusing on specific sports rather than on the overall practice of sports) and to replicate the analyses for a variety of sports (for a better generalizability of the findings). We therefore examined four categories of sports requiring (possibly or necessarily) facilities and investigated separately their relationship with the spatial accessibility to the corresponding facilities.

Moreover, previous studies of such relationships have often relied on measures of densities defined within administrative areas [20] or on measures of spatial accessibility from the residence based on Euclidean measures [9,11,12]. Relatively few studies have relied on measures of spatial accessibility that take the street network into account [14,16-19]. Moreover, studies have assessed either densities or distances to the closest facility but not both. In constrast, the present study relies on two complementary measures of spatial accessibility, both taking into account the street and road network: the street distance to the closest facility and the count of facilities in street network buffers centered on the residence.

While studies often rely on an a priori hypothesis of a reasonable distance of access to facilities (in order to reduce the number of hypotheses tested and statistical tests performed), it may be possible to obtain information from the data on the distance beyond which a given facility is not considered as spatially accessible. Such information would be of major usefulness for policy makers in their work of planning of the location of resources. We therefore performed a sensitivity analysis to derive information on the distance beyond which a facility is located too far to be effectively used.

Finally, various studies have shown that different characteristics of the neighborhood of residence are related to physical activity [21,22], including socioeconomic characteristics [23,24]. To examine whether these neighborhood characteristics confound the relationships of interest between the spatial accessibility to sport facilities and sport practice, the latter associations were examined before and after adjustment for these neighborhood variables.

Overall, the purpose of this study was to investigate the associations between the spatial accessibility to sport facilities and the practice of the corresponding activities in the Paris metropolitan area, after carefully controlling for various individual socio-demographic characteristics and neighborhood socioeconomic and urbanicity variables that may confound the associations.

## Methods

### Population

The RECORD Cohort Study (“Residential Environment and CORonary heart Disease”, http://www.record-study.org) was used for the analyses [25]. As previously described, 7290 participants were recruited between March 2007 and February 2008 [26-29]. The participants benefitted from a free medical checkup offered every 5 years by the French National Health Insurance System for Salaried Workers to all working and retired employees and their families. Participants were recruited without a priori sampling during these 2-hour–long preventive checkups conducted by the Centre d’Investigations Préventives et Cliniques in 4 of its health centers, located in the Paris metropolitan area (Paris, Argenteuil, Trappes, and Mantes-la-Jolie). Eligibility criteria were as follows: age 30 to 79 years; ability to complete study questionnaires; and residence in one of the 10 (out of 20) administrative divisions of Paris or 111 (out of 1301) other municipalities of the metropolitan area selected a priori. Of the persons selected for participation, 83.6% accepted to participate and completed the data collection protocol. A previous study showed that, due to the absence of random sampling, our sample was not representative of the population of the municipalities from which it was drawn: people with a high education level, people living closer from the examination centers involved in the recruitment, and people living in affluent neighborhoods and in low density neighborhoods had higher rates of participation in the study [30].

All participants underwent a physical examination, completed questionnaires, and were geocoded with accuracy based on their residential address in 2007–2008. Research assistants rectified all incorrect or incomplete addresses with the participants by telephone. Extensive investigations with local Departments of Urbanism were conducted to complete the geocoding. Spatial coordinates and geographic codes of the street, block, and block group were searched for each participant. Precise coordinates and block-group codes were identified for 100% of the participants. The study protocol was approved by the French Data Protection Authority, which supervises any use of personal health data in France and provides ethical allowances for the processing of these data in biomedical research.

### Measures

#### Outcomes

From a list of activities, the participants were asked to report the sport and recreational activities they had performed over the previous 7 days, and where they had practiced such activities, i.e., in their neighborhood, out of their neighborhood or both (within and outside the neighborhood). The participants were asked to rely on their subjective perception of their neighborhood to locate these activities (neither participants were provided objective indications on the size of the neighborhood to consider, nor were they asked to objectify how they perceived it). From these data, we grouped a number of sport activities into four categories to create outcome variables: team sports, racket sports, sports requiring a swimming pool, and fitness. Team sports included soccer, basketball, handball, hockey, rugby, and volleyball. Racket sports referred to tennis, squash, badminton, and ping pong. Activities requiring a swimming pool were swimming and aquaerobics. Fitness included aerobics, weight training, cardio training, and gymnastics. Two binary variables were created for each category of sports: one indicating whether the individuals had practiced that particular activity over the previous 7 days and one indicating whether they had performed at least part of the activity within their neighborhood rather than only out of their neighborhood.

#### Individual adjustment covariates

Several socio-demographic characteristics were considered: age, sex, individual education, marital status, occupation, household income, homeownership, financial strain, and Human Development Index of each participant’s country of birth.

Age was divided in 3 classes (30–44, 45–59, and 60 years or older). Education was divided in 4 classes: no education; primary education and lower secondary education; higher secondary education and lower tertiary education; and upper tertiary education. Marital status was coded in 2 classes: living alone or as a couple. Occupation was coded in 4 categories: high white-collar workers, intermediate occupations, low white-collar workers, and blue-collar workers. Ownership of dwelling was coded as a binary variable. Household income adjusted for household size was divided into 4 categories. We attributed to each individual the 2004 Human Development Index (HDI) of his/her country of birth [31], as a crude proxy of his/her cultural origin. Following the United Nations Development Program, a categorical variable was used to distinguish people born in low-development countries (HDI < 0.5), in medium-development countries (HDI between 0.5 and 0.8), in France, and in other high-development countries (HDI > 0.8).

#### Weather variables

We used daily meteorological data provided by Meteo France for 2007–2008. Data from 5 or 6 meteorological stations (according to the weather parameter considered) were averaged to derive daily information on a regional scale. Based on these data, we defined average weather variables for each participant for the recruitment day and 7 previous days. Several daily meteorological parameters were considered: minimum temperature; maximum temperature; average temperature; rainfall; wind speed; time of sunshine; presence of fog or not; and presence of mist or not. All the resulting variables were divided in 4 categories based on the quartiles.

#### Assessment of sports facilities

An exhaustive list of sport facilities present over the study territory and their characteristics and geographic coordinates as of December 2008 was obtained from the Census of Sport Facilities established by the Ile-de-France Regional Direction of Youth, Sports, and Social Cohesion (Direction Régionale de la Jeunesse, des Sports et de Cohésion Sociale d’Ile-de-France, DRJSCS). The Census of Sport Facilities is a fully exhaustive database of all facilities and sites allowing sport activities that are open to the public for free or not [13].

**Figure 1:**
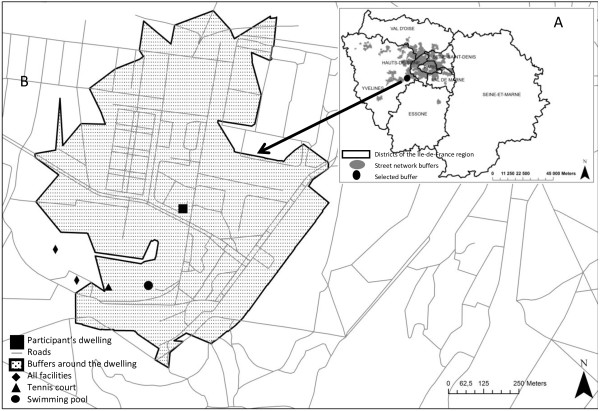
**Methods of determination of spatial accessibility to sport facilities in the study. A.** is a representation of the Ile-de-France region. Based on road and street network data from the National Geographic Institute, the shortest distance between each respondent’s home and the closest sport facility of each category, and the number of facilities around each respondent’s home in 1 km street network radius buffers were estimated (**B**). The 1 km street network buffers for all the participants are shown in grey in **A**.

A geographic Information System (GIS) was used to develop indices of spatial accessibility to the recreational facilities of interest for the present study. Based on road and street network data from the National Geographic Institute, the ArcInfo GIS software and its Network Analyst were used to estimate the shortest distance between each respondent’s home and the closest sport facility, and the number of facilities around each respondent’s home in 1 km street network radius buffers [4,32]. Separate spatial accessibility variables were determined for each of the 4 sport categories investigated. The distances were then coded into 2 categories: distance >1 km vs. distance <1 km and the number of facilities spatially accessible were coded into 3 categories: 0 facility, 1 facility, 2 or more facilities (see Figure 1). Moreover, for a sensitivity analysis on the exact distance that matters to access to facilities, we defined a series of binary variables indicating whether the distance to the closest facility of each type was less than 500, 600, 700, 800, 900, 1000, 1100, 1200, 1300, 1400, or 1500 m.

#### Other neighborhood variables

We also took into account neighborhood factors that are susceptible to confound the associations between the presence of facilities and the practice of the corresponding sports [12].

Regarding socioeconomic factors, we considered neighborhood education (proportion of residents aged > 15 years with an upper tertiary education from the 2006 census), neighborhood income (median income per consumption unit, 2006 Tax Registry of General Directorate of Taxation), and neighborhood mean real estate prices (mean value of dwellings sold in 2003–2007, Paris Notaries). Neighborhood population density, as a demographic variable, was also considered (2006 population census). Finally, we took into account the density of destinations (number of services accessible in the neighborhood).

All these variables were determined within 1 km street network buffers centered on the participants’ building of residence. Neighborhood variables were divided into 4 categories comprising a similar number of individuals, to assess dose–response associations without forcing linearity.

### Data analysis

To account for geographic autocorrelation in each outcome, we estimated multilevel logistic regression models with participants nested within IRIS administrative neighborhood (the median number of residents in 2006 in these neighborhoods was 2536, interquartile range: 2161, 3115). The statistical analyses involved several steps (each time for the 4 outcomes of sport practice over the previous 7 days):

1. We estimated models including only age and sex as explanatory variables to assess variability in each outcome between IRIS neighborhoods.

2. We then included the other individual adjustment covariates and the weather variables into the models.

3. Associations of the spatial accessibility to facilities with sport practice were tested by adding to the models obtained in step 2 either the distance to the nearest facility or the number of facilities in the 1 km street network radius neighborhoods (the potential neighborhood confounders were not included at this step of the analyses). As a sensitivity analysis on the distance beyond which a facility may be considered as more difficult to use, separate models including each a spatial accessibility variable with a different distance threshold were compared.

4. We then tested one by one the other neighborhood characteristics (related to the socio-demographic and service environment), in models adjusted for individual and weather covariates (each neighborhood variable in a separate model).

5. Finally, we progressively combined into one model the spatial accessibility variables and the other contextual variables that were independently associated with each outcome (one of the aims was to assess whether the associations between the accessibility to facilities and sport practice identified in step 3 persisted after adjustment for other neighborhood variables).

All models were estimated with Markov chain Monte Carlo simulation using WinBUGS 1.4.3.21 [33].

## Results

Overall, fitness was the most prevalent type of sports among the categories examined, as practiced by 17.3% of the participants over the previous 7 days. Regarding the other sport categories, 10.0% of the participants had performed an activity requiring a swimming pool, 5.5% a racket sport, and 3.6% had practiced a team sport (Table 1).

**Table 1:** Prevalence of sport activities over the previous 7 days, the RECORD Study, 2007-2008

**Activities**	**n**	**Prevalence**
**Team sports**	**250**	**3.6**%
Basketball	25	0.3%
Football (soccer)	191	2.6%
Handball	6	0.1%
Hockey	3	0.04%
Rugby	15	0.2%
Volleyball	22	0.3%
**Racket sports**	**376**	**5.5**%
Tennis	247	3.4%
Squash	32	0.4%
Badminton	58	0.8%
Ping pong	63	0.9%
**Activities requiring a swimming pool**	**728**	**10.0**%
Swimming	701	9.6%
Aquaerobics	30	0.4%
**Fitness**	**1099**	**17.3**%
Aerobics	296	4.1%
Cardio training	6	0.1%
Weight training	359	4.9%
Gymnastics	600	8.2%

### Relationship between individual variables and sports practice

As shown in Table 2, simultaneously considering the different individual socio-demographic factors, the odds of practicing a team sport in the previous 7 days increased with the level of human development index of the country of birth. Moreover, the odds of using a swimming pool, the odds of practicing a racket sport, and the odds of fitness exercise in the previous 7 days increased both with the level of education of participants and with household income (Table 2). Regarding weather variables, a high minimum temperature increased the odds of using a swimming pool whereas a high minimum temperature decreased fitness practice. No other weather variables were associated with the outcomes.

**Table 2:** Associations between individual characteristics and sport practice, the RECORD Study, 2007-2008

**Variables**	**Team sports**^**b **^**(n = 7290) OR**^**a **^**(95% CrI)**	**Racket sports**^**b **^**(n = 7290) OR**^**a **^**(95% CrI)**	**Swimming**^**c **^**OR**^**a **^**(n = 7290)(95% CrI)**	**Fitness**^**c **^**(n = 7290) OR**^**a **^**(95% CrI)**
Men (vs. women)	13.00 (7.12 – 26.98)	2.39 (1.78 – 3.26)	0.75 (0.63 – 0.89)	0.58 (0.50 – 0.68)
Age (vs. 30–44)				
45–59	0.36 (0.27 – 0.48)	0.76 (0.59 – 0.96)	0.86 (0.71 – 1.03)	0.87 (0.74 – 1.03)
60–79	0.15 (0.08 – 0.26)	0.51 (0.36 – 0.70)	0.82 (0.66 – 1.03)	1.36 (1.14 – 1.63)
Living alone (vs. as a couple)	0.78 (0.56 – 1.08)	0.55 (0.41 – 0.73)	1.26 (1.06 – 1.51)	1.09 (0.93 – 1.27)
Individual education (vs. no education)				
Medium-low education	1.32 (0.78 – 2.30)	2.37 (1.08 – 6.12)	1.37 (0.88 – 2.19)	1.53 (1.08 – 2.23)
Medium-high education	1.39 (0.81 – 2.46)	3.09 (1.42 – 7.96)	1.89 (1.22 – 3.00)	2.05 (1.45 – 2.98)
High education	1.20 (0.68 – 2.19)	4.11 (1.88 – 10.63)	1.90 (1.22 – 3.06)	2.06 (1.44 – 3.01)
Occupation (vs. blue-collar workers)				
Low white-collar workers	0.65 (0.44 – 0.96)	1.01 (0.62 - 1.70)	1.23 (0.87 - 1.78)	1.13 (0.85 - 1.54)
Intermediate occupations	0.72 (0.40 –1.28)	1.41 (0.77 - 2.62)	1.38 (0.88 - 2.19)	1.16 (0.78 - 1.73)
High white-collar workers	0.69 (0.43 – 1.11)	1.06 (0.64 - 1.84)	1.27 (0.88 - 1.88)	1.04 (0.76 - 1.44)
Perceived financial strain	1.06 (0.73 – 1.51)	0.70 (0.44 – 1.08)	0.78 (0.59 – 1.03)	0.90 (0.71 – 1.13)
Household income (vs. low income)				
Medium-low income	1.05 (0.72 – 1.52)	1.69 (1.13 – 2.57)	1.58 (1.20 – 2.08)	1.55 (1.24 – 1.95)
Medium-high income	0.86 (0.56 – 1.31)	1.84 (1.22 – 2.84)	1.69 (1.27 – 2.26)	1.49 (1.17 – 1.91)
High income	0.68 (0.42 – 1.10)	1.99 (1.31 – 3.12)	1.65 (1.23 – 2.22)	1.74 (1.36 – 2.23)
Homeownership (vs. non ownership)	1.18 (0.87 – 1.60)	1.16 (0.91 – 1.50)	1.11 (0.92 – 1.33)	1.28 (1.09 – 1.49)
Human Development Index of country of birth (vs. born in France)				
Low	1.57 (0.90 – 2.62)	0.63 (0.28 – 1.23)	0.66 (0.39 – 1.08)	1.19 (0.82 – 1.69)
Medium	1.60 (1.12 – 2.28)	0.76 (0.52 – 1.09)	0.86 (0.66 – 1.11)	0.91 (0.73 – 1.13)
High (other than France)	1.95 (1.27 – 2.91)	0.70 (0.43 – 1.07)	0.87 (0.64 – 1.15)	1.01 (0.79 – 1.28)
Minimum temperature (vs. low)				
Mid-low	–	–	1.27 (1.00 – 1.61)	0.72 (0.60 – 0.87)
Mid-high	–	–	1.49 (1.19 – 1.88)	0.79 (0.66 – 0.95)
High	–	–	1.60 (1.28 – 2.01)	0.80 (0.67 – 0.96)

^a^ OR: Odds ratio, 95% CrI: 95% Credible Interval.

^b^ Models adjusted for age, sex, marital status, individual education, occupation, homeownership status, perceived financial strain, household income, and the level of human development of the country of birth.

^c^ Models further adjusted for minimum temperature.

### Associations between spatial accessibility to sports facilities and the practice of sports

**Figure 2:**
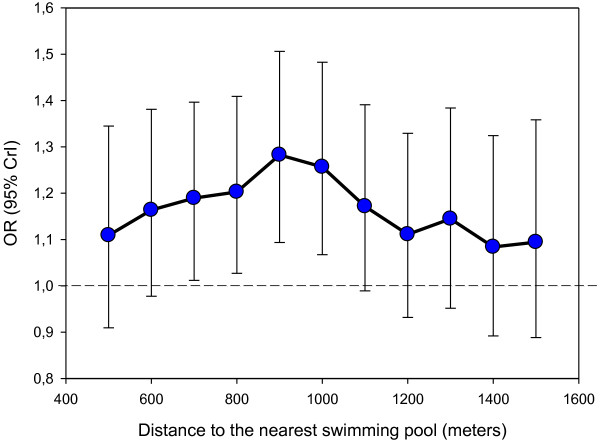
**Relationship between the distance to the closest swimming pool and the practice of the corresponding sports.** Each point in the Figure corresponds to a separate model with a binary variable for distance determined with a different cutoff, adjusted for individual and weather variables.

As shown in Table 3, only the spatial accessibility to a swimming pool was associated with the practice of related activities (mostly swimming) over the previous 7 days, after adjusting for individual socioeconomic and weather covariates. The probability of such a practice was higher when the distance to the nearest pool was less than 1 km. As shown in Figure 2, a detailed sensitivity analysis indicated that the strength of the relationship between a binary variable for distance to the closest swimming pool and the practice of a related activity increased from 500 m (as a cutoff for the distance variable) to 1000 m and then decreased after, suggesting that the distance effect was best captured when a cutoff of 1000 m was used. Furthermore, these analyses suggest that an increase in the odds of using a swimming pool was only detected when there were two or more swimming pools in the 1 km radius residential buffer.

**Table 3:** Associations between spatial accessibility to sport facilities and the practice of sports, the RECORD Study, 2007-2008

**Variables**	**Team sports**^**b **^**(n = 7290) OR**^**a **^**(95% CrI)**	**Racket sports**^**b **^**(n = 7290) OR**^**a **^**(95% CrI)**	**Swimming**^**c **^**(n = 7290) OR**^**a **^**(95% CrI)**	**Fitness**^**c **^**(n = 7290) OR**^**a **^**(95% CrI)**
Distance to the nearest facility <1 km (vs. > 1 km)	1.17 (0.76 – 1.88)	1.05 (0.81 – 1.38)	1.26 (1.07 – 1.48)	1.01 (0.85 – 1.21)
Density of facilities (vs. no facility)				
One facility	1.64 (0.94 – 2.95)	0.82 (0.55 – 1.22)	1.12 (0.88 – 1.42)	0.94 (0.75 – 1.19)
Two facilities or more	1.22 (0.78 – 2.00)	1.18 (0.82 – 1.56)	1.30 (1.09 – 1.55)	1.06 (0.88 – 1.19)

^a^ OR: Odds ratio, 95% CrI: 95% Credible Interval.

^b^ Models adjusted for age, sex, marital status, individual education, occupation, homeownership status, perceived financial strain, household income, and the level of human development of the country of birth.

^c^ Models further adjusted for minimum temperature.

No effect of spatial accessibility was detected for the other 3 groups of sports (team sports, racket sports, and fitness). However, when the practice of sports in one’s (perceived) residential neighborhood was examined as the outcome rather than the overall practice of sports, the presence of a facility at less than 1 km from the residence was associated with the odds to practice in one’s residential neighborhood both a racket sport and activities related to a swimming pool.

### Associations between the other contextual variables and the practice of sports

Few associations were identified (Table 4). The probability of practice of a racket sport and fitness in the previous 7 days increased with neighborhood income, after taking into consideration the individual socioeconomic covariates. Moreover, the probability of practice of a team sport was lower when the overall density of services in the area of residence was high. However, none of the contextual factors was related to swimming (which outcome is therefore not reported in Table 4). The observed relationship between the spatial accessibility to a swimming pool and the practice of swimming or related activities (Table 3) was therefore not confounded by the other neighborhood variables examined.

**Table 4:** Associations between contextual variables and the practice of sports, the RECORD Study, 2007-2008

**Variables**	**Team sports**^**b **^**(n = 7290) OR**^**a **^**(95% CrI)**	**Racket sports**^**b **^**(n = 7290) OR**^**a **^**(95% CrI)**	**Fitness**^**c **^**(n = 7290) OR**^**a **^**(95% CrI)**
Density of destinations (vs. low)			
Mid-low	0.80 (0.57 – 1.13)	–	–
Mid-high	0.69 (0.48 – 0.99)	–	–
High	0.48 (0.32 – 0.73)	–	–
Neighborhood income (vs. low)			
Mid-low	–	2.05 (1.38 – 3.12)	1.17 (0.95 – 1.45)
Mid-high	–	2.56 (1.75 – 3.87)	1.21 (0.98 – 1.49)
High	–	2.41 (1.61 – 3.67)	1.47 (1.19 – 1.83)

^a^ OR: Odds ratio, 95% CrI: 95% Credible Interval.

^b^ Models adjusted for age, sex, marital status, individual education, occupation, home ownership status, perceived financial strain, household income and the level of human development of the country of birth.

^c^ Model further adjusted for minimum temperature.

## Discussion

We examined associations between the spatial accessibility to sport facilities measured with different indicators and the practice of sport activities, after ensuring the correspondence between specific sport facilities and related sports, and controlling for individual socio-demographic variables and contextual variables.

These findings suggest that (i) the spatial accessibility to swimming pools was associated with the practice of the corresponding sports; and that (ii) other contextual factors allowed the identification of neighborhoods where specific sports are less likely to be practiced.

### Study limitations and strengths

The main strength of the present study is the correspondence that we were able to establish between indicators of spatial accessibility to facilities and the practice of sports, and the fact that four categories of sports were separately examined. Other strengths of the study include the large sample accurately geocoded over the Paris Ile-de-France region; the exhaustive and high-quality Census of Sport Facilities that allowed us to assess the type and location of sports facilities; the fact that we measured the spatial accessibility to facilities with two complementary approaches that took into account the street network (distance to the nearest facility and count of facilities around the dwelling [4]); and the examination of neighborhood factors that may confound the associations between the spatial accessibility to facilities and the practice of the corresponding sports.

However, a major study limitation is that the RECORD population is not strictly representative of the Paris metropolitan region, even if we a priori selected a panel of municipalities from the region to ensure the presence of people from all socioeconomic backgrounds. As noted in the Methods section, neighborhood factors affecting the odds of participation in the RECORD Study were identified [30]. In an attempt to reduce the magnitude of bias, these neighborhood characteristics were taken into account for the adjustment of the relationships of interest in the present study. Another limitation is that the findings were based on cross-sectional data, not allowing us to demonstrate that the relationships between spatial accessibility to facilities or neighborhood variables and sport practice are attributable to a causal effect of the former on the latter. Our data do not allow us to rule out confounding due to selective residential migration (people willing to practice a particular sport may choose to live nearby the corresponding facilities). Finally, we were unable to take into account the transportation modes available to the participants as a potential modifier of the relationships of interest between the spatial distance to sport facilities and the use of such facilities.

### Study findings

Independent effects of individual education and household income were found for the practice of racket sports and fitness and for the use of a swimming pool, while none of these factors were related to the practice of collective sports. Socioeconomic status has often been found to be associated with the level of physical activity [21,34,35]. An interest of the present study is to demonstrate independent associations of two individual socioeconomic indicators with the practice of various (but not all) sports. Regarding household income, cost is an established barrier to healthy behaviors in general and it is known that the cost of access to sport facilities can be a barrier to their use [36-38]. This is confirmed in our French sample by the association with household income for 3 of the 4 categories of sports investigated (for all categories except team sports because people commonly have free access to soccer fields for example). However, for these 3 categories of sports, the associations with individual education were stronger than those with household income, suggesting that in addition to costs, a number of intrapersonal or cognitive variables also significantly matter. Our interpretation is that a higher education level is related to a better knowledge of benefits or risks associated with behaviors, to a more accurate knowledge of health recommendations, to a higher priority given to health, to more positive attitudes towards health promotion, and possibly to a greater ability to convert intentions to comply with health recommendations into action [39]. A previous study of ours already documented strong individual education effects on the practice of jogging [40].

This study revealed that the probability of swimming or practicing a related sport was associated with the spatial accessibility to a swimming pool. The combination of our sensitivity analysis on the distance to the closest swimming pool and of our analysis of the count of swimming pools accessible in the immediate neighborhood allowed us to characterize in a very precise way the spatial accessibility effect. The sensitivity analysis is based on the idea that the use of a too short or too long radius for the spatial accessibility variable would result in the dilution of the association of interest. The approach indicated that the association was the strongest when accessibility to a swimming pool was measured within 900 m or 1000 m from the residence. An interpretation is, for example, that spatial accessibility to swimming pools within 1500 m leads to a weaker association because it also includes swimming pools located too far away to be regularly used. Thus the sensitivity analysis indicates that swimming pools located within 900–1000 m are those which most optimally (though weakly) increase the probability of utilization. This finding is coherent with the idea that 1000 m correspond to a 10 to 15 minute walk in an urban setting and encompass the easily accessible resources [19,41,42].

No spatial accessibility effect, however, was documented for the other categories of sports examined. A possible explanation is that team sports, racket sports, and fitness can be practiced without a facility (in private or public gardens or open spaces for example) while swimming requires access to a pool. Another explanation for the lack of association between the spatial accessibility to facilities and the practice of team or racket sports may be that these sports require the coordination among multiple people. People may have to cover longer distances to practice such sports because they also have to take into account the spatial accessibility to the facility for their partners.

A previous publication based on data of the Ile-de-France region found that the type of sport facilities spatially accessible depended on the urbanicity degree and on the degree of socioeconomic disadvantage of the neighborhoods [13]. However, our findings suggest that, in the relatively well served Ile-de-France region, such disparities in the spatial accessibility to sport facilities do not have a major impact on utilization, except perhaps for swimming pools.

The study was based on the assumption that beyond spatial access to sports facilities, various contextual characteristics may affect the practice of sports. It seemed useful to include these factors in the analysis to detect possible territorial disparities in practices and to examine whether the association identified between the spatial accessibility to a pool and the corresponding practice persisted after taking into consideration these multiple factors. An important finding is that 2 out of the 4 sports examined (racket sports and fitness) were practiced less frequently in socially disadvantaged neighborhoods, after adjustment for individual socioeconomic characteristics. The present study does not directly indicate whether residents of disadvantaged neighborhoods have a lower overall practice of sports, or whether they practice different sports, but the individual and contextual effects documented here suggest that the former alternative may be true.

## Conclusions

Overall, this study found that the spatial accessibility to swimming pools was associated with swimming and related activities. This study also shows that other residential neighborhood characteristics, especially socioeconomic advantage, were associated with the practice of specific sports. This article provides baseline estimates of spatial access to and use of sport facilities in the Ile-de-France region and of the relationships between the two, to orientate the promotion of physical activity. Strategies to increase participation in physical activities should include improving the spatial access to specific facilities (which may be particularly relevant in less urbanized regions than Ile-de-France) and improving the financial access to many of these facilities (by reducing the costs of access to private but also public facilities). The educational disparities in sport practice that persist after taking into account the geographical and financial accessibility components also warrant promotion efforts.

## Abbreviations

RECORD: Residential Environment and CORonary heart Disease; OR: Odds ratio; 95% CrI: Credible Interval; HDI: Human Development Index.

## Competing interests

The authors declare that they have no competing interests.

## Authors’ contributions

NK contributed to design the specific study, performed all the statistical analyses, and led the drafting of the manuscript. BC supervises the RECORD research project and contributed to the design of the specific study. All authors contributed to the interpretation of the results and to the review of the manuscript drafts. They have all read and approved the final manuscript.
